# Ultrasound-derived changes in thickness of human ankle plantar flexor muscles during walking and running are not homogeneous along the muscle mid-belly region

**DOI:** 10.1038/s41598-019-51510-4

**Published:** 2019-10-21

**Authors:** E. F. Hodson-Tole, A. K. M. Lai

**Affiliations:** 1 Research Centre Musculoskeletal Science and Sports Medicine, Department of Life Sciences, Manchester Metropolitan University, Manchester, UK; 2 Department Biomedical Physiology and Kinesiology, Simon Fraser University, Burnaby, Canada

**Keywords:** Skeletal muscle, Image processing

## Abstract

Skeletal muscle thickness is a valuable indicator of several aspects of a muscle’s functional capabilities. We used computational analysis of ultrasound images, recorded from 10 humans walking and running at a range of speeds (0.7–5.0 m s^−1^), to quantify interactions in thickness change between three ankle plantar flexor muscles (soleus, medial and lateral gastrocnemius) and quantify thickness changes at multiple muscle sites within each image. Statistical analysis of thickness change as a function of stride cycle (1d statistical parametric mapping) revealed significant differences between soleus and both gastrocnemii across the whole stride cycle as they bulged within the shared anatomical space. Within each muscle, changes in thickness differed between measurement sites but not locomotor condition. For some of the stride, thickness measures taken from the distal-mid image region represented the mean muscle thickness, which may therefore be a reliable region for these measures. Assumptions that muscle thickness is constant during a task, often made in musculoskeletal models, do not hold for the muscles and locomotor conditions studied here and researchers should not assume that a single thickness measure, from one point of the stride cycle or a static image, represents muscle thickness during dynamic movements.

## Introduction

Measures of skeletal muscle thickness, taken from static images, are an important indicator commonly used to infer strength or muscle mass^1^. Yet, muscle thickness dynamically changes as a function of activation^2^ and contraction^3^. These changes in thickness are also important, considering muscle’s isovolumetric properties and geometric relationships between fascicle lengths and pennation in the context of muscle gearing^4,5^. Measuring muscle thickness is therefore important for quantifying and inferring key muscle properties to improve understanding of dynamic muscle mechanics. However, while muscle thickness is recognised as an important variable to measure, how muscles change thickness during dynamic tasks such as locomotion is not well understood.

In studies of human skeletal muscles, ultrasound imaging provides a well-tolerated, minimally invasive means of visualising the muscles that can be used across a wide range of motor tasks (e.g. posture^6^, isokinetic dynamometer tasks^7^ and locomotion^8^). Typically, such studies focus on the behaviour of an individual muscle and, where measures of thickness are taken, they are typically from the mid-region of the recorded images and simplify the aponeurosis to being a straight line (e.g. a line between two points defined at each end of the image). Such measures are then used to describe the behaviour of the whole muscle. Skeletal muscles are not however simple shapes and, in many body regions, changes in their shape may be constrained by the properties and behaviour of muscles that share the anatomical compartment^9^. Whether a single measure of muscle thickness represents the region of muscle belly imaged and how thickness changes occur simultaneously across groups of synergistic muscles has therefore not previously been reported.

In recent years there has been an increase in the availability of automated or semi-automated computational techniques for the analysis of ultrasound images that can quantify a range of skeletal muscle properties^10–16^. These computational approaches not only enable objective assessment of collected images but, due to reduced operator demand, also enable larger data sets to be assessed and more measures to be taken with concomitant improvements in reproducibility. To quantify muscle thickness, active shape model approaches have been proposed to identify the aponeurosis boundaries of all muscles within the image^11^. The approach enables measures of thickness to be recorded at multiple points across the image and from multiple muscles within the image, with no prior assumptions of their shape(s) (i.e. straight aponeuroses). The purpose of the work presented here is therefore to: (i) identify whether a single measure of muscle thickness, in the central portion of the image, can be considered representative of a whole muscle region and; (ii) quantify changes in thickness of synergistic muscles sharing an anatomical compartment (i.e. the calf region of the human lower leg) during different locomotor tasks. Specifically, we report thickness changes in human soleus (SO), medial and lateral heads of gastrocnemius (MG, LG) muscles, determined from active shape model based analysis of ultrasound images recorded during walking and running at a range of steady-state speeds.

## Methods

### Data collection

Data were collected as part of a wider study, the details of which have been reported previously^8^. Briefly, ten participants (one female; mean ± S.D. age 27 ± 6 years, height 1.81 ± 0.08 m, mass 80.2 ± 11.7 kg) were recruited to the project, which had been approved by the local human research ethics committee at University of Queensland where data were collected (Ref. No.: 2012001215). Participants were included if they were free from any recent or pre-existing musculoskeletal injury that would likely affect his/her ability to complete the exercise protocol. Participants provided written informed consent, and the work was completed in accordance with the principles laid down by the *Declaration of Helsinki*.

An instrumented treadmill (Tandem, Advanced Mechanical Technology Inc., Watertown, Massachusetts, USA) was used to record ground reaction forces (2000 Hz sampling frequency) while each participant walked (0.7, 1.4 and 2.0 m s^−1^) and ran (2.0, 3.0, 4.0 and 5.0 m s^−1^) whilst wearing minimalist shoes (Xero Shoes, Boulder CO, USA). Participants self-selected their gait kinematics. All walked with a rear foot strike pattern. Three ran with rear foot and three with fore foot strike patterns, while four participants ran with a rear foot strike pattern for the slower running speeds and switched to fore foot strike pattern for the fastest speed.

Ultrasound image sequences were recorded (~80 frames s^−1^, Echoblaster 128, Telemed, Lithuania) using a 60 mm long linear transducer (LV7.5/60/128Z-2, Telemed, Lithuania, 7 MHz latent frequency) secured to the right leg using elasticised bandage. Unlike the commonly seen ‘T’ shaped transducers, this transducer is flat with the cable exiting at one end which helps to prevent it moving during dynamic tasks. The transducer cable was orientated so that it trailed up the participant’s back, to the pelvis then across to the ultrasound device box, situated alongside the treadmill. With the transducer laid flat on the skin, elasticated bandage was tightly applied to minimise movement relative to the skin, particularly for the faster running conditions. The bandage completely covered the transducer, overlapping the edges minimising any potential for the probe to rock during locomotion (see Supplementary Fig. S1).

Each gait condition was completed twice, once with the transducer secured over the mid-belly region of MG and once with the transducer over the mid-belly region of LG. The mid-belly region is typically the thickest portion of each muscle, and provides data that are considered representative of the whole muscle mechanics^17^. In each case, the location of the transducer was selected to provide a clear view of the aponeuroses of the muscles of interest (MG or LG and SO), aligned to the fascicle plane, as is common in many ultrasound derived *in vivo* muscle mechanics studies^3,6,10,16^. The time taken to transfer the transducer also ensured participants rested between trials to reduce the potential effects of fatigue on data collection. Ultrasound image sequences were synchronised with ground reaction force data via a TTL pulse sent from the ultrasound device at the onset of image capture. Data were recorded for 15 seconds in each condition. This trial duration was used in part to minimise potential fatigue related to accumulation of exercise duration across the full experimental protocol.

### Ultrasound image analysis

**Figure 1:**
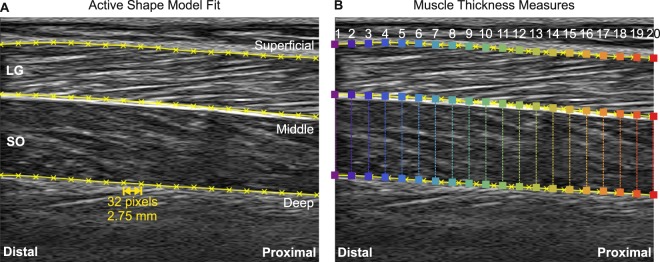
(**A**) Example ultrasound image of lateral gastrocnemius and soleus muscles, with each of the aponeuroses fit with the active shape model (yellow). The fit provided 19 points along each aponeurosis, separated by 32 pixels (2.75 mm). (**B**) Representation of the 20 point landmarks distributed along each aponeurosis shape model fit (coloured squares). Coloured vertical lines represent thickness measures recorded in soleus. The colour scheme is used in following figures to indicate the distal-proximal location of reported thickness measures.

To identify the location of the deep and superficial aponeuroses of each muscle in recorded ultrasound images three aponeurosis boundaries: (i) superficial; (ii) middle and; (iii) deep, were identified by segmenting each image using an active shape model approach^11^ (Fig. 1A and see Supplementary Videos 1–3). The process involved creating a points distribution model (PDM) for each participant and muscle view, by manually labelling every 20^th^ frame of ultrasound image sequences recorded during running at 3 and 4 m s^−1^. The PDM captured the shape variations the aponeuroses of that participant underwent for the given ultrasound view (i.e. medial or lateral). The two running trials were selected for this purpose as they represented conditions across which large differences in shape were seen.

Labels for each of the three aponeurosis boundary lines consisted of the two dimensional locations (x- and y-coordinate) of 19 points, each separated by 32 pixels (~2.75 mm) so the line spanned the entire image width (Fig. 1A and see Supplementary Videos 1–3). The number of training images ranged between 12 and 20 across participants and muscle views, depending on the number of images captured for the labelled trial. The mean shape and covariance matrix were calculated from these data, and the eigenvectors and eigenvalues of the covariance matrix calculated to provide a model of shape variation, whilst preserving 98.5% of the data set’s original variance.

In the second phase of the training process the PDM is augmented with Gaussian models of image intensity at each landmark, to allow a probabilistic search for known shapes in analysed images. As described by Darby and colleagues^11^, and following the methods of Cootes and Taylor^18^, a normalised intensity gradient along a straight line extending 2 pixels either side of a given landmark and perpendicular to the PDM contour was sampled for every landmark across the full set of training images. The samples for each landmark are then modelled by a (2 pixel +1)-dimensional Gaussian distribution. This process was repeated at three lower image resolutions, to provide a multiresolution active shape model (ASM) facilitating a coarse-to-fine search for solutions in analysed images (See Fig. A1 in 11).

To automatically segment images the multiresolution ASM adjusts the set of PDM landmarks to accurately describe the aponeuroses in the image being analysed. The process is initialised by taking the fit from the previous frame and applying it to the current frame. The position of each landmark is adjusted, from this initial position, along a straight line extending perpendicular to the neighbouring landmarks (Fig. 1). At each new position, the normalised intensity value is determined and its likelihood, given the pre-trained intensity model, is calculated. This is done by calculating the Mahalanobis distance, which is proportional to the log of the probability that the new normalised sample was generated by the Gaussian distribution describing intensities for that landmark. Once the Mahalanobis distribution is minimised for every landmark, the new shape configuration is compared against the PDM derived model of shape variation and the nearest plausible shape of the aponeurosis is found by shifting to within three standard deviations of the model variables.

For the first frame of each trial there is no prior shape from which the search may begin and a different initialisation approach is therefore required. For this initialisation two additional image resolutions were added to the ASM training procedure (to give a total of five resolution levels), to allow larger landmark adjustments to occur at the start of the search process. Two separate searches were then started, one for each set of training images (i.e. one for each series of images from the two hand labelled trials). Each of these searches was initialised with the mean shape from that trial’s hand labelled images. From the two resulting shapes the one with the lowest Mahalanobis distance across all landmarks and image resolutions was selected to start the search of frame one of the trial being analysed.

The segmentation procedure was applied to all the trials (including the hand labelled trials) of each participant, using the PDM and ASM developed from their images. Unlike the work presented Darby *et al*.^11^ images were therefore segmented using training data from that person’s own trials, an approach we found provided the best fits given the large range of aponeurosis shapes found across the analysed data set. The resulting segmentations were visually inspected to check the aponeuroses had been appropriately fit. Data from both ultrasound views of one participant were removed, as the aponeuroses were not clear enough to confidently confirm the accuracy of the fitting procedure. Therefore, in total, segmentation data from over 66,000 images recorded from nine participants across the seven gait conditions were taken forward for analysis.

The resulting fits were interpolated and the vertical distance between 20 equally spaced points along the deep and superficial aponeurosis of each muscle were calculated to represent muscle thickness (Fig. 1B). Together with the mean of these thickness values, this calculation was done for each frame of each trial. Images recorded with the transducer over MG were used to provide thickness measures of MG, whilst those recorded with the transducer over LG were used to provide thickness measures of LG and SO. Thicknesses from four consecutive strides of each trial were identified on the basis of the recorded ground reaction force data^8^. These thickness values were interpolated to 200 data points, representing 100% stride cycle. Thickness change was calculated by subtracting the thickness measured during a static, quiet standing trial and saved for statistical analysis.

### Statistical analysis

One-dimensional statistical parametric mapping (SPM) was used to determine whether thickness differed between any of the 20 muscle regions measured and whether there was an effect of gait/speed condition across the analysed strides in each muscle (MATLAB implementation from the open-source spm1d software package available at http://www.spm1d.org/Downloads.html). A two way ANOVA with repeated measures was employed, with dependent factor thickness and fixed factors muscle region (1–20 and the mean) and gait condition (7 × walk/run conditions), repeated for four strides. Where statistically significant differences were identified (*p* ≤ 0.05) a two-tailed paired t-test, with *Bonferroni* correction was used to identify their location. To identify whether muscle regions differed from the mean, and to minimise the number of repeated tests completed, comparisons were made between the mean and each of four points selected from the mid-image region (Fig. 1B).

One-dimensional SPM was also used to determine whether changes in thickness differed between the three muscles analysed. Two way ANOVA with repeated measures was again used, this time with fixed factors muscle (SO, MG, LG) and gait/speed condition, with stride as the repeat factor. The mean thickness was used as the dependent factor. Where significant differences were identified (*p* ≤ 0.05) two-tailed paired t-tests, with *Bonferroni* correction were again used to identify their location.

## Results

### Thickness measures across different muscle regions

**Figure 2:**
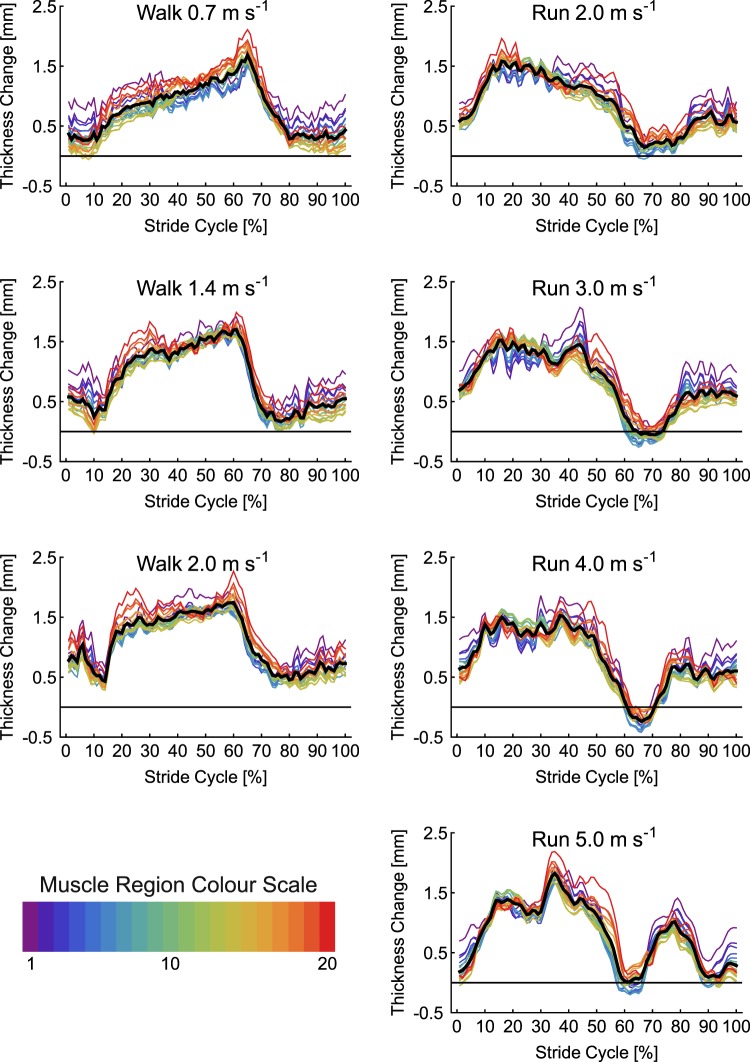
Change in thickness of soleus muscle a function of stride cycle for each of the seven locomotor conditions studied. Twenty coloured lines represent the change in thickness at each point along the length of the muscle (see colour scale bar and Fig. 1). The thick black line represents the mean value. Thickness change was calculated as the thickness subtracted from the thickness recorded during a quiet standing trial.

**Figure 3:**
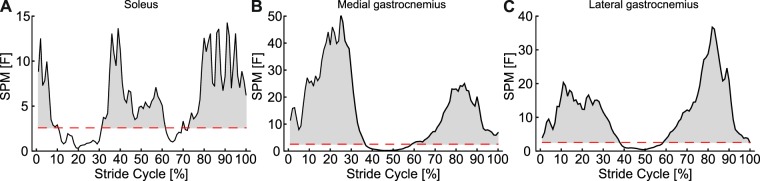
SPM ANOVA results for (**A**) soleus, (**B**) medial gastrocnemius and (**C**) lateral gastrocnemius, showing where significant differences in thickness at different muscle regions occur as a function of stride cycle

**Figure 4:**
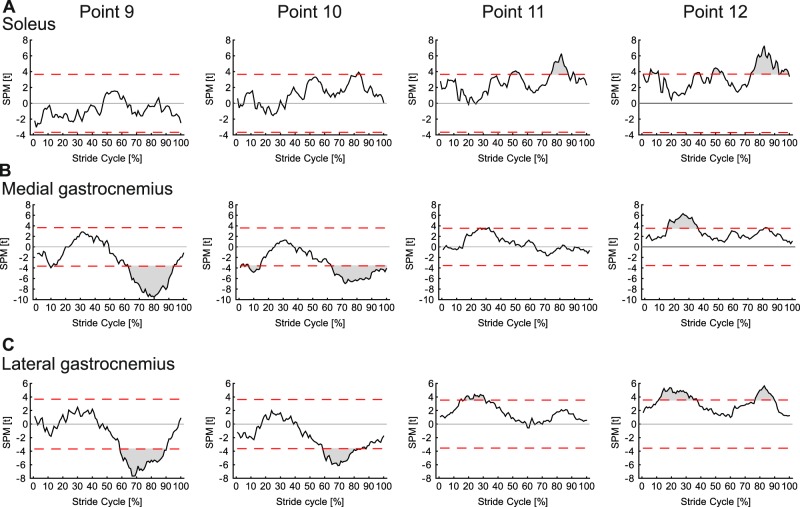
SPM paired t-test results comparing thickness muscle values at different central image points to the mean thickness. Columns represent the different muscle region comparisons for each row: (**A**) soleus, (**B**) medial gastrocnemius, (**C**) lateral gastrocnemius. See Fig. 1 for point positions in ultrasound image.

Changes in SO muscle thickness for each of the seven conditions are shown in Fig. 2. A general pattern of increased thickness over the first 50% of the stride cycle, followed by rapid thinning of the muscle was seen across all gait conditions. Across the gait conditions the maximum difference in thickness between the 20 muscle regions was 1.31 mm. SPM analysis revealed no significant effect of condition on thickness (critical *F* = 4.90, maximum *F* = 3.31, mean ± S.D. *F* = 1.60 ± 0.69) and no interaction with muscle region (critical *F* = 1.52, maximum *F* = 0.69, mean ± S.D. *F* = 0.40 ± 0.13). There were however significant differences in thickness between regions over three periods: 1–10%, 32–61% and 70–100% stride cycle (Fig. 3A). Across the gait conditions the mean difference in thickness across SO regions was 0.53 ± 0.07 mm, with a maximum difference of 1.05 mm occurring at 8% of 1.4 m s^−1^ walking stride. Post hoc comparison of thickness values from the centre of the image to the mean thickness showed no significant difference between the mean compared to points 9 and 10, short periods of differences did occur when comparing points 11 and 12 to the mean (Fig. 4A).

**Figure 5:**
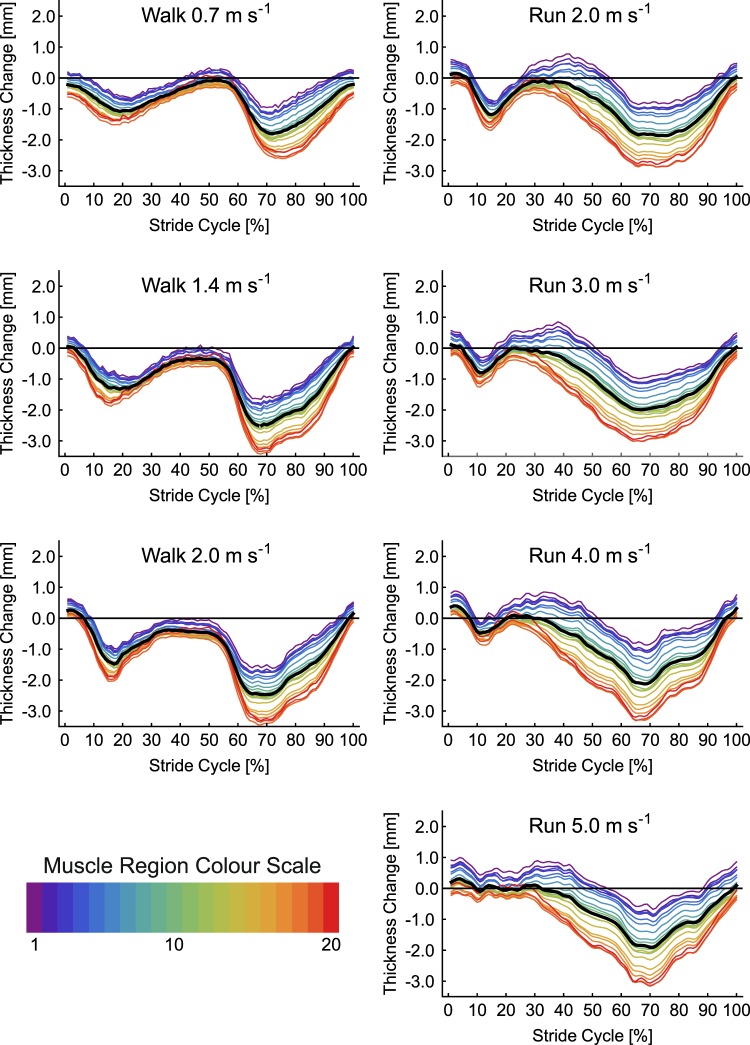
Change in thickness of medial gastrocnemius muscle a function of stride cycle for each of the seven locomotor conditions studied. Twenty coloured lines represent the change in thickness at each point along the length of the muscle (see colour scale bar and Fig. 1). The thick black line represents the mean value.

Changes in MG thickness for each of the seven conditions are shown in Fig. 5. Two periods of MG thinning followed by thickening were evident in each condition, although the first period was shorter at the faster gait speeds. There was a much larger difference in thickness values than observed in SO, particularly during the swing phase of the running conditions. SPM analysis found no significant differences in thickness between conditions (critical *F* = 4.82, maximum *F* = 2.06, mean ± S.D. *F* = 1.08 ± 0.46) and no interaction with muscle region (critical *F* = 1.52, maximum *F* = 1.26, mean ± S.D. *F* = 0.77 ± 0.18). Significant differences in thickness at different muscle regions occurred during two periods of the stride cycle: 1–36% and 59–100% (Fig. 3B). Across the gait conditions the mean difference in thickness across MG regions was 1.34 ± 0.44 mm, with a maximum difference of 2.61 mm occurring at 69% of 5.0 m s^−1^ running stride. Post-hoc comparison of thickness values from the centre of the image to the mean thickness found significant differences in all points, except point 11 (Fig. 4B).

**Figure 6:**
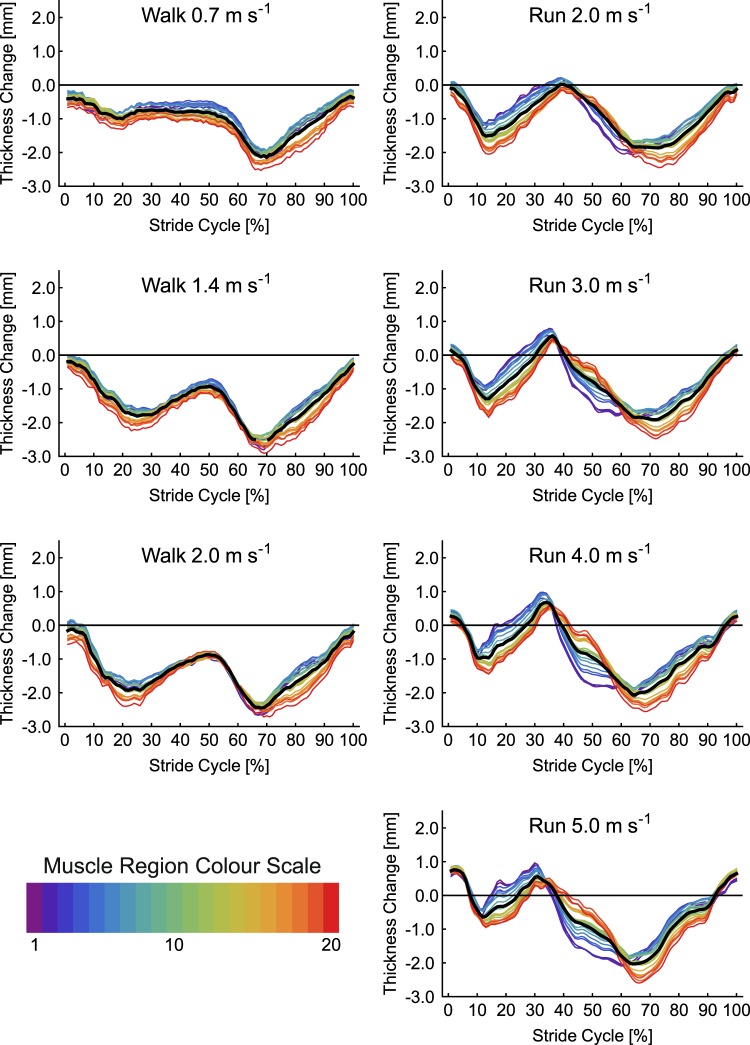
Change in thickness of lateral gastrocnemius muscle a function of stride cycle for each of the seven locomotor conditions studied. Twenty coloured lines represent the change in thickness at each point along the length of the muscle (see colour scale bar and Fig. 1). The thick black line represents the mean value.

Changes in LG thickness for each of the seven conditions are shown in Fig. 6. As in MG two periods of thinning and thickening were apparent, with the first period becoming shorter in running compared to walking. During the first 40% of the stride cycles for running the distal portion of the muscle thickened most. In the subsequent period (~40–60% stride) this region then thinned more than other regions. SPM analysis again found no effects of condition on thickness (critical *F* = 4.83, maximum *F* = 3.80, mean ± S.D. *F* = 1.83 ± 0.88) and no interaction with muscle region (critical *F* = 1.52, maximum *F* = 1.13, mean ± S.D. *F* = 0.56 ± 0.17). Significant differences in thickness between muscle regions were found, spanning two periods of the stride cycle: 1–37% and 59–100% (Fig. 3C). Across the gait conditions the mean difference in thickness across LG regions was 0.67 ± 0.17 mm, with a maximum difference of 1.54 mm occurring at 48% of 4.0 m s^−1^ running stride. Post hoc analysis comparing thickness values in the centre of the image to the mean, found significant differences at each of the four points measured (Fig. 4C).

### Comparison of changes in thickness across the Triceps surae muscle group

**Figure 7:**
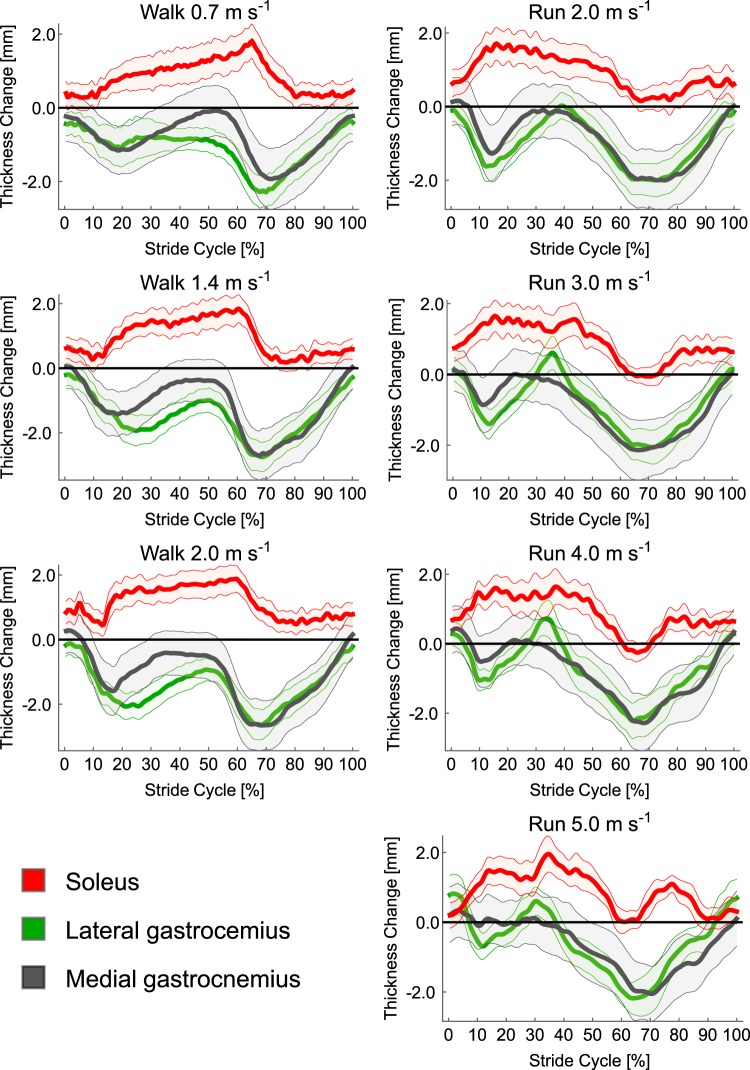
Mean (thick line) and S.E.M. (thin line, colour in-fill) thickness of each muscle (soleus = red; medial gastrocnemius = grey; lateral gastrocnemius = green) as a function of stride cycle for each of the locomotor conditions. SPM paired t-test showed that soleus thickness changes were significantly different to both medial gastrocnemius and lateral gastrocnemius for the whole stride cycle. SPM paired t-test showed that soleus thickness changes were significantly different to both medial gastrocnemius and lateral gastrocnemius for the whole stride cycle.

The mean change in thickness (i.e. mean of the 20 thickness measures) of each of the muscles across each of the seven gait conditions are shown in Fig. 7. The pattern of thickness change was strikingly different in SO compared to LG and MG, with SO becoming thicker over the stride cycle and both MG and LG thinning. The patterns of thickness change in LG and MG were very similar, an observation confirmed by statistical analysis. Specifically, SPM found no significant differences in mean thickness of the three muscles between conditions (critical *F* = 4.82, maximum *F* = 2.75, mean ± S.D. *F* = 1.49 ± 0.58). There was a significant difference however between muscles that persisted for the whole stride cycle (critical *F* = 12.25, maximum *F* = 115.33, mean ± S.D. *F* = 48.85 ± 27.74). A significant interaction also occurred between condition and muscle, although this only occurred over 29–31% stride cycle. Post hoc analysis showed no significant difference between MG and LG (critical *t* =  ±3.27, minimum *t* = −2.93, mean ± S.D. *t* = −1.55 ± 0.72). Significant differences were found between SO and both MG and LG, both of which occurred over the full stride cycle (SO-MG: critical *t* =  ±3.42, maximum *t* = 16.08, mean ± S.D. *t* = 13.37 ± 2.37; SO-LG: critical *t* =  ±3.42, maximum *t* = 17.53, mean ± S.D. *t* = 12.60 ± 2.73).

## Discussion

### Can a single measure of muscle thickness be considered representative of a whole muscle region

The initial aim of the presented work was to determine whether a single, ultrasound based, measure of muscle thickness, taken from the mid-region of the image, can be considered representative of the portion of muscle imaged over the course of a stride cycle. The results show that across muscle regions, differences in muscle thickness can occur in SO, MG and LG (Fig. 3). While the absolute differences may seem small, when considered relative to the resting thickness of the muscles (MG ~21 mm; LG ~18 mm: SO ~15 mm, taken from the quiet standing trial; see also^19^) the differences found are quite large (~7–14%). To outline the potential effects of this, we consider an example SO muscle with 44 mm long fascicles and 15 mm belly thickness. Varying thickness by 15% would alter pennation from ~20° to ~23°, or if pennation were held constant fascicle length would increase to ~51 mm. The changes in thickness that occur over the stride cycle show that researchers should not assume that a single assessment of thickness obtained at one point of the stride cycle or from a static image (i.e. image taken whilst participant maintains a specified pose) represents the muscle thickness during dynamic movements. Assumptions that muscle thickness is constant during a task, often made in musculoskeletal models, therefore do not hold for the muscles and locomotor conditions studied here.

The use of computational image segmentation makes it possible to determine muscle thickness for different regions of the muscle (Fig. 1 and see Supplementary Videos 1–3), and hence researchers have the opportunity to either determine the mean thickness for the imaged portion or sub-select and determine thickness for specific regions of interest. Computational image analysis tools may not however be an option for all researchers, meaning that manual digitisation is likely to be required. In such instances the results presented here suggest that single measures of thickness, taken from the middle portion of the image, may be representative of the whole muscle during the first 50% of gait cycle (Fig. 4). Equally, taking measures within a 64 pixel wide region, distal to the image midline can provide measures that do not differ significantly from the mean. Greater significant differences were seen in the results for MG and LG compared to SO, suggesting that in these muscles greater caution is needed when selecting the location at which thickness will be measured.

As our analysis is based on a single data set, it is not clear how sensitive these recommendations may be to differences in ultrasound transducer positioning over the muscle. It is likely that exact positioning of the transducer differs between research groups and will also be influenced by participant characteristics and the motor task(s) studied. However, the general practice of placing the transducer over the mid-belly muscle region and aligning it to the fascicle plane is a common protocol reported in the majority of work exploring human muscle mechanics during dynamic tasks^3,6,10,16^. While our recommendations for the image regions from which thickness measures should be taken may not be directly transferable to other research groups, these results provide evidence of the potential sensitivity of thickness measures to the image/muscle region analysed.

### Patterns of thickness change in muscles sharing an anatomical compartment

The second aim of the work presented was to investigate the patterns of thickness change in the three ankle plantar flexor muscles for different locomotor conditions. SO, MG and LG all share the posterior compartment of the shank and a common tendon of insertion. Thus changes in shape of one muscle must interact with the space occupied by the others and be modulated by length changes imposed through interactions with their common tendon.

The first portion of the stride cycle in all conditions was characterised by thickening of SO and thinning of MG and LG, suggesting that the muscles are working or bulging against each other. Each muscle is typically actively contracting during this portion of the stride cycle, therefore this interaction could be modulated by the amplitude of the activation, given the associations between activation level and intramuscular pressure^20^ and muscle stiffness^21^. In addition, bulging of the muscles and changes in stiffness could also influence the gearing values seen in each muscle^4^. Thickness change of adjacent muscles, sharing an anatomical compartment, likely imposes additional constraints on the muscle shape changes^9^ and could therefore be an additional factor to be considered for understanding mechanisms of muscle gearing.

### Study limitations

We have previously noted that the results presented here are based on analysis of a single data set, recorded in one laboratory. Recommendations of the optimum image region from which to assess muscle thickness during dynamic tasks may therefore not translate directly to other data sets. It is also important to note that securing the ultrasound transducer onto the leg will inherently constrain movement of the superficial surface of both MG and LG muscles, a factor that has been shown to influence skeletal muscle mechanics^22,23^. This is an unavoidable problem, given that the transducer must be secure to enable capture of the required images. However, it does mean that MG and LG thickness changes reported here are almost entirely determined by displacement of their deep aponeuroses. This means we likely underestimate the changes in thickness of these muscles during walking and running and may also have influenced the relationships seen with change in thickness of SO.

## Conclusions

The ankle plantar flexor muscles of humans show dynamic changes in muscle thickness during walking and running at a range of steady-state speeds. Although our understanding of such relationships, derived using ultrasound imaging, will be influenced by methods of securing the transducer to the limb, we observed interactions between LG, MG and SO as the muscles bulge within the anatomical compartment they share. Furthermore, muscle thickness is not constant along the muscle length imaged, with dissimilar changes in thickness occurring in different regions of the muscle across the stride. However, for some portions of the stride, thickness measures taken from the distal-mid image region represented the mean muscle thickness and may therefore be a reliable region from which measures should be taken.

## Supplementary information

Supplementary Information S1

Supplementary Video Material 1

Supplementary Video Material 2

Supplementary Video Material 3

## Data Availability

Muscle thickness values are available on DRYAD for readers to access: 10.5061/dryad.crjdfn30n.
