# Supplementary material for: Ultrasound-derived changes in thickness of human ankle plantar flexor muscles during walking and running are not homogeneous along the muscle mid-belly region
Source: Sci Rep. 2019 Oct 21;9:15090. doi: 10.1038/s41598-019-51510-4 (PMC6803718; doi:10.1038/s41598-019-51510-4)
Supplement: Supplementary file 1 — Supplementary Information S1 [file 41598_2019_51510_MOESM1_ESM.pdf]

Ultrasound-derived changes in thickness of human ankle plantar flexor muscles during walking and running are not homogeneous along the muscle mid-belly region

E.F. Hodson-Tole and A.K.M. Lai

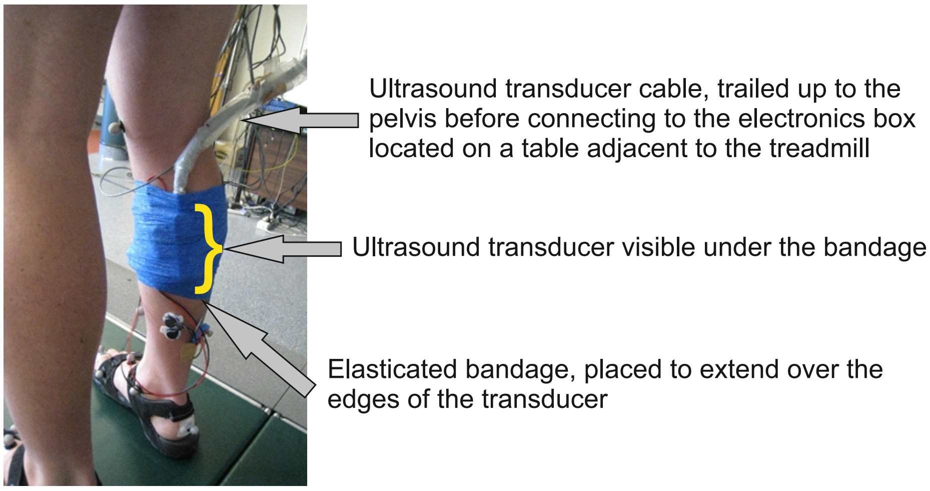

Figure S1. Image taken from one participant showing the placement of the ultrasound transducer over the medial gastrocnemius muscle and the bandaging used to secure the transducer over the limb. Note that a flat ultrasound transducer (LV7.5/60/128Z-2, Telemed, Lithuania) was used for data collection, to ensure it could be held securely in place on the leg and minimise movement.
