# Supplementary figures and images for: Ultrasound-derived changes in thickness of human ankle plantar flexor muscles during walking and running are not homogeneous along the muscle mid-belly region
Source: Sci Rep. 2019 Oct 21;9:15090. doi: 10.1038/s41598-019-51510-4 (PMC6803718; doi:10.1038/s41598-019-51510-4)

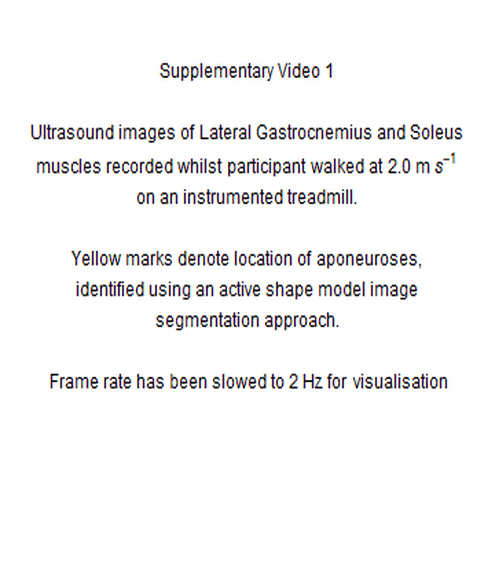

Supplement: Supplementary file 2 — Supplementary Video Material 1 [file 41598_2019_51510_MOESM2_ESM.gif]

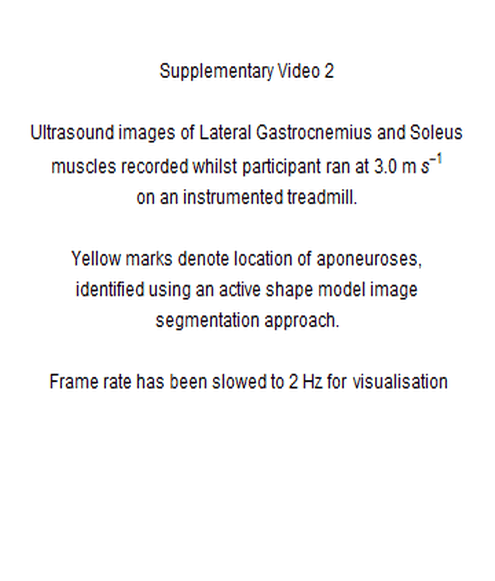

Supplement: Supplementary file 3 — Supplementary Video Material 2 [file 41598_2019_51510_MOESM3_ESM.gif]

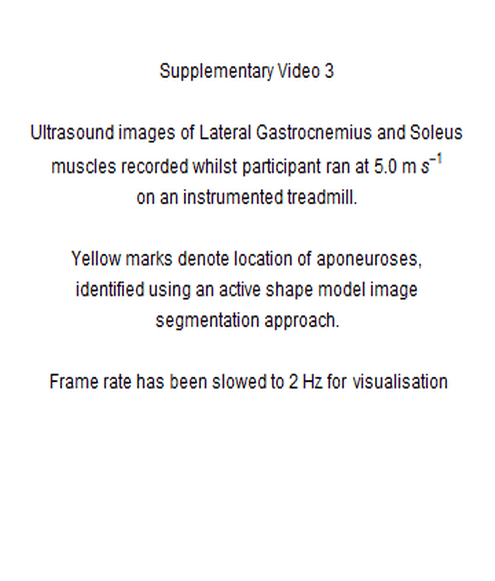

Supplement: Supplementary file 4 — Supplementary Video Material 3 [file 41598_2019_51510_MOESM4_ESM.gif]
